# Vitamin D supplementation in diabetic retinopathy: an optical coherence tomography angiography study of changes in macular perfusion

**DOI:** 10.1186/s40942-026-00822-5

**Published:** 2026-02-24

**Authors:** Sahba Fekri, Maryam Hosseini, Golnar Hassanzadeh, Hosein Nouri, Sare Safi, Nooshin Ahmadi, Zahra Khorrami, Seyed-Hossein Abtahi

**Affiliations:** 1 Ophthalmic Research Center, Research Institute for Ophthalmology and Vision Science, Shahid Beheshti University of Medical Sciences, Tehran, Iran; 2 Department of Ophthalmology, Labbafinejad Medical Center, Shahid Beheshti University of Medical Sciences, Tehran, Iran; 3 School of Medicine, Tehran University of Medical Sciences, Tehran, Iran; 4 Ophthalmic Epidemiology Research Center, Research Institute for Ophthalmology and Vision Science, Shahid Beheshti University of Medical Sciences, Tehran, Iran; 5 Research Institute for Endocrine Sciences, Shahid Beheshti University of Medical Sciences, Tehran, Iran; 6 Research Institute for Ophthalmology and Vision Science, Shahid Beheshti University of Medical Sciences, Tehran, Iran

**Keywords:** Diabetic retinopathy, Vitamin D, Optical coherence tomography angiography, Diabetic macular ischemia

## Abstract

**Background:**

Observational studies suggested a link between vitamin D deficiency (VDD) and diabetic retinopathy (DR). However, evidence from interventional studies on possible effects of VDD correction on retinal microvasculature in DR is scarce.

**Methods:**

This single-arm exploratory interventional study included 60 eyes from 30 patients with VDD and type 2 diabetes mellitus (DM), without DR or with mild or moderate non-proliferative DR (NPDR). Participants received oral vitamin D_3_ (50,000 IU/week for 8 weeks) followed by a daily maintenance dose (800 IU). Optical coherence tomography angiography (OCTA) imaging was performed before and three months after vitamin D repletion. Outcome measures were macular perfusion parameters and best-corrected visual acuity (BCVA). Linear mixed effects models were used to evaluate the association between changes in outcome measures with change in serum 25(OH)D_3_ levels.

**Results:**

The mean serum 25(OH)D_3_ level increased significantly post-intervention (14.20 ± 4.04 to 55.82 ± 6.79 ng/mL; *p* < 0.001), and VDD was corrected in all patients. A small improvement was noted in BCVA with marginal statistical significance (0.12 ± 0.13 to 0.10 ± 0.13 Logarithm of the Minimum Angle of Resolution; *p* = 0.051). Other than a small reduction in parafoveal superficial vessel density at the superior quadrant (-3.23 ± 6.66%, *p* = 0.011), none of the perfusion metrics had changed three months after VDD correction. Linear models yielded no association between changes in serum 25(OH)D_3_ levels and alterations in BCVA or OCTA macular perfusion metrics.

**Conclusions:**

Based on this non-controlled interventional study, short-term effects of VDD correction in people with DM without severe DR does not appear to significantly influence the visual acuity or macular perfusion profile on OCTA.

## Introduction

Diabetic retinopathy (DR) is a major microvascular complication of diabetes mellitus and a leading cause of vision loss, affecting nearly 40% of diabetic patients worldwide; in 2020, approximately 4.4 million individuals experienced blindness or moderate-to-severe visual impairment due to DR [1, 2]. DR is characterized by microvascular dysfunction, neurodegeneration, and inflammation, driven by oxidative stress, free radical production, and chronic inflammatory pathways [3]. Hyperglycemia induces intracellular oxidative stress through glucose metabolism, auto-oxidation, and advanced glycation end product formation, while oxidative damage may persist despite adequate glycemic control [4]. Dysregulation of inflammatory mediators results in increased expression of vascular endothelial growth factor (VEGF) and other cytokines, leading to retinal microvascular alterations such as microaneurysms, capillary non-perfusion, vascular leakage, and neovascularization [5]. Advances in optical coherence tomography angiography (OCTA) have revealed that these microvascular changes precede clinically apparent DR, which progresses from non-proliferative stages (NPDR) to proliferative disease (PDR) with vision-threatening complications [6]. Current treatment options are typically employed at later stages, when irreversible changes to the retinal vasculature are more pronounced. These therapies often fail to address the underlying causes of microvascular damage and require frequent, invasive interventions over long periods [7]. These limitations highlight the need for early, adjunctive treatments targeting the underlying pathophysiological mechanisms to slow disease progression and improve visual outcomes.

Observational Studies have explored the potential link between vitamin D deficiency (VDD) and DR, with some suggesting an inverse association between vitamin D status and the risk of developing DR [8–13]. However, the evidence on the causal role of vitamin D in DR remains inconclusive. More recently, large Mendelian randomization analyses using genome-wide association study data have failed to demonstrate a causal effect of vitamin D levels on DR, despite observational associations [14–16]. These findings underscore the ongoing controversy about whether vitamin D plays a direct role in the pathogenesis of DR or if observed associations may reflect confounding by other factors, such as lifestyle or metabolic conditions.

Some interventional studies have suggested potential benefits in improving retinal outcomes when vitamin D levels are normalized [17, 18]. These findings are not yet robust enough to draw definitive conclusions regarding vitamin D as a therapeutic intervention for DR.Vitamin D is linked to various non-skeletal effects due to the presence of vitamin D receptors (VDR) in a diverse range of cells throughout the body [19, 20] and its potential role in DR can be explained by its ability to modulate cell-microenvironment interactions, such as regulating mitochondrial function, immunoregulation, and processes like autophagy, apoptosis, angiogenesis, and epithelial-mesenchymal transition [21–23]. Despite this biological plausibility, data from randomized controlled trials on the direct impact of vitamin D supplementation on the retina in diabetic patients are scarce, and some studies have failed to demonstrate significant effects on retinal perfusion or visual acuity [24, 25]. Given the existing uncertainties, the impact of vitamin D supplementation on the retinal microvasculature in individuals with DR remains under investigation.

In this study, we aimed to assess early changes in OCTA macular perfusion parameters following the correction of vitamin D deficiency in patients with DM and without proliferative or severe non-proliferative DR.

## Methods

### Design

This prospective interventional study assessed the impact of correcting vitamin D deficiency (VDD) on macular perfusion in patients with type 2 diabetes mellitus without severe non-proliferative or proliferative diabetic retinopathy. Ethical approval was obtained from the Ophthalmic Research Center Ethics Committee at Shahid Beheshti University of Medical Sciences (Ethics approval code: IR.SBMU.MSP.REC.1400.833). The study was conducted in accordance with the principles of the Declaration of Helsinki. Written informed consent was obtained from all individual participants prior to enrollment. Participants were recruited from patients referred to the tertiary Retina and Vitreous Clinic of Labbafinejad Hospital, affiliated with Shahid Beheshti University of Medical Sciences (Tehran, Iran), between April 2021 and December 2022.

### Study population and eligibility criteria

Eligibility criteria were having: (i) at least 18 years of age, (ii) type 2 DM without severe NPDR or macular edema, and (iii) laboratory-confirmed VDD, i.e., serum 25(OH)D_3_ level below 20 ng/mL. Patients were excluded if they (i) had undergone any previous ocular surgery within the preceding six months, except for an uncomplicated cataract surgery, (ii) had hypercalcemia or hyperphosphatemia, or (iii) were taking vitamin D supplements at the time of recruitment. Patients who were non-adherent to the supplementation regimen or those in whom hypovitaminosis D was not corrected after two months of supplementation were excluded from the analysis. Both eyes of each eligible individual were enrolled in this study.

### Patient assignment and interventions

All recruited eyes underwent a complete ophthalmic examination at the beginning of the study. Parameters including age, sex, blood pressure, Hb1Ac levels, best-corrected visual acuity (BCVA), and intraocular pressure (IOP) were measured. A complete history of DM and associated medications was also obtained. Further, a detailed fundus examination was performed using a non-contact 78 D or 90 D lens to check for the presence and severity of DR. The first OCTA session was scheduled before vitamin D supplementation initiation. Then, patients received a pearl of vitamin D_3_ (D-Vigel 50,000 IU, Daana Pharmaceutical Company, Iran) once a week for eight consecutive weeks as the only intervention of the study. After supplementation, serum 25(OH)D3 levels were measured. Once vitamin D levels were normalized (serum 25[OH]D₃ > 30 ng/mL), participants continued on a maintenance regimen of 800 IU daily. Three months after VDD was corrected, a second ophthalmic examination and OCTA imaging were performed, followed by an evaluation of changes in OCTA indices, IOP, and BCVA. The primary outcome of the study was the change in OCTA-derived macular perfusion parameters from baseline to three months after vitamin D repletion. The secondary outcome was the change in BCVA over the same follow-up period.

BCVA was assessed via Snellen chart examination by a trained optometrist and was converted to Logarithm of the Minimum Angle of Resolution (LogMAR) values by convention. IOP was measured by the Goldmann applanation tonometer. An ophthalmologist checked for and graded the DR severity using three field fundus photographs (optic disk centered, fovea centered, and centered on the temporal edge of the macula) following the International DR Severity Scale [26].

### OCTA imaging

OCTA images were acquired by an RTVue XR 100 Avanti instrument (Optovue, Inc., Fremont, CA, USA) set on the macular protocol (6 mm × 6 mm, centered on the fovea). Only images with a quality of 5/10 and above and minimal artifacts were assessed. The software automatically measured all parameters and calculated vessel density (VD), representing the percentage of pixels corresponding to blood flow within vessels. The superficial vessel density (SVD) was set at the inner limiting membrane (ILM), and 9 μm above the inner plexiform layer (IPL), and the deep vessel density (DVD) was set between 9 μm above the IPL and 9 μm below the outer plexiform layer (OPL).

The fovea was defined as the central 1 mm diameter circle. Foveal avascular zone (FAZ) was defined as the avascular area at the center of fovea without visible vessels on OCTA. The parafoveal and perifoveal areas were the 1–3 mm and 3–6 mm annuli surrounding the fovea, respectively.

### Statistical analysis

Because of the limited prior evidence on the magnitude of OCTA changes following vitamin D repletion, a formal sample size calculation was not performed. The sample size was based on feasibility, and the study should therefore be considered exploratory and hypothesis-generating. A descriptive summary of numerical (mean, standard deviation [SD], interquartile range [IQR], and range) and categorical (frequency and percentage) variables was generated. The Wilcoxon signed-rank test was used for inter-visit comparison of measurements, considering the non-normal distribution of continuous variables per the Shapiro-Wilk test results. P-values from multiple comparisons were adjusted for the false discovery rate using the Benjamini–Hochberg procedure. As this rank-based method recalculates p-values according to their relative order, identical adjusted p-values may occur when multiple tests yield similar unadjusted values.

The association between changes in outcome measures and changes in serum 25(OH)D_3_ was evaluated using linear mixed effects models. For each outcome measure, three levels of adjustment were set. First, a model took into account only the baseline value of that measure and the intra-patient correlation between pairs of eyes. A second model additionally controlled for age and gender. A third model took into account other possible patient-level confounding factors as well, i.e., DM duration, type of DM treatment, HbA1c level, systolic blood pressure, and retinopathy stage at baseline. All tests were two-tailed, and p-values < 0.05 were considered significant. Data analysis was performed using R statistical software version 4.1.2 (R Foundation for Statistical Computing, Vienna, Austria).

## Results

### Baseline characteristics

Sixty eyes from 30 individuals, whose demographic and clinical information are summarized in Table 1, were included. Sixteen (53.3%) patients had no clinically evident diabetic retinopathy (NDR), 7 (23.3%) had mild non-proliferative DR (NPDR), and 7 (23.3%) had moderate NPDR. The retinopathy stage remained unchanged in all participants throughout the study, except one patient with mild NPDR who progressed to moderate NPDR. Disease duration was shorter than 10 years in 16 patients (53.3%). Most patients (73.3%) were taking oral medications only, and the rest received insulin. Table 1 summarizes the rest of the relevant clinical information.

**Table 1:** Patient-level demographic and clinical information

Characteristic	Mean ± SD/ Frequency (%)
Age (years)	58.5 ± 10.2
Gender (females)	13 (43%)
DM duration (years)	10.4 ± 7.2
Hemoglobin A1c (%)	6.7 ± 1.0
Systolic blood pressure (mmHg)	118.2 ± 9.1
Diastolic blood pressure (mmHg)	74.1 ± 5.6

### Postinterventional changes

Baseline VDD (mean ± SD: 14.20 ± 4.04) was corrected in all patients, resulting in a mean post-supplementation serum 25(OH)D_3_ level of 55.82 ± 6.79 (*p* < 0.001). Table 2 provides a comparison of OCTA metrics before and after vitamin D supplementation among the entire study population. A small trend toward improvement was seen in BCVA values, but its statistical significance was marginal (0.12 ± 0.13 to 0.10 ± 0.13 LogMAR, *p* = 0.051). None of the OCTA metrics showed any significant change compared to baseline (*p* > 0.05), except for parafoveal SVD at the superior quadrant, which was slightly reduced (-3.23 ± 6.66%, adjusted p-value = 0.011) (Table 2).

**Table 2:** Changes in eye-level parameters after vitamin D deficiency correction (*N* = 60 eyes)

Parameter	Baseline	After Three Months	Difference	Unadjusted *P*-value	Adjusted *p*-value
FAZ area (mm^2^)	0.30 ± 0.11	0.32 ± 0.13	0.01 (0.08)	0.363 ^α^	0.959 ^α^
FAZ perimeter (mm)	2.17 ± 0.48	2.19 ± 0.57	0.02 (0.34)	0.493 ^α^	0.959 ^α^
Foveal SVD (%)	16.94 (6.97)	15.88 (6.17)	-1.08 (5.1)	0.108	0.959
Foveal DVD (%)	32.27 (8.11)	29.85 (9.25)	-2.48 (7.24)	**0.010**	0.190
Parafoveal DVD (%)	50.04 (4.16)	49.82 (4.76)	-0.22 (4.59)	0.714	0.959
Parafoveal SVD (%)	46.91 (4.87)	46.73 (5.59)	-0.14 (5.43)	0.789 ^α^	0.959 ^α^
Parafoveal superior hemiretina SVD (%)	46.68 (4.97)	46.73 (5.7)	0.09 (5.92)	0.806 ^α^	0.959 ^α^
Parafoveal superior hemiretina DVD (%)	50.23 (4.54)	49.98 (5.33)	-0.26 (5.03)	0.684	0.959
Parafoveal inferior hemiretina SVD (%)	47.24 (5.00)	46.76 (5.94)	-0.48 (5.52)	0.504	0.959
Parafoveal inferior hemiretina DVD (%)	49.54 (4.38)	49.66 (4.73)	0.11 (4.97)	0.865	0.959
Parafoveal temporal quadrant SVD (%)	46.53 (6.14)	46.83 (6.36)	0.31 (6.06)	0.806 ^α^	0.959 ^α^
Parafoveal temporal quadrant DVD (%)	51.35 (4.61)	50.92 (5.54)	-0.42 (4.95)	0.510	0.959
Parafoveal superior quadrant SVD (%)	50.92 (5.54)	47.69 (6.14)	-3.23 (6.66)	**< 0.001**	**0.011**
Parafoveal superior quadrant DVD (%)	49.25 (5.14)	49.07 (5.42)	-0.2 (5.61)	0.776	0.959
Parafoveal nasal quadrant SVD (%)	45.86 (5.15)	46.47 (6.01)	0.64 (6.06)	0.420	0.959
Parafoveal nasal quadrant DVD (%)	50.85 (5.19)	51.57 (4.81)	0.75 (5.77)	0.317	0.959
Parafoveal inferior quadrant SVD (%)	48.10 (4.8)	47.41 (6.17)	-0.66 (5.6)	0.579 ^α^	0.959 ^α^
Parafoveal inferior quadrant DVD (%)	48.06 (5.06)	48.2 (5.74)	0.1 (5.96)	0.927 ^α^	0.959 ^α^
Perifoveal DVD (%)	45.09 (5.6)	45.49 (5.68)	0.31 (6.43)	0.706	0.959
Perifoveal SVD (%)	47.04 (4.15)	47.21 (4.24)	0.2 (4.71)	0.739	0.959
Perifoveal superior hemiretina SVD (%)	46.81 (4.24)	46.91 (4.24)	0.12 (4.64)	0.687 ^α^	0.959 ^α^
Perifoveal superior hemiretina DVD (%)	45.70 (5.71)	45.99 (6.38)	0.18 (7.19)	0.880 ^α^	0.959 ^α^
Perifoveal inferior hemiretina SVD (%)	47.37 (4.5)	47.44 (4.42)	0.11 (4.93)	0.523 ^α^	0.959 ^α^
Perifoveal inferior hemiretina DVD (%)	44.50 (5.76)	44.87 (5.63)	0.3 (6.39)	0.719	0.959
Perifoveal temporal quadrant SVD (%)	43.02 (4.89)	42.99 (4.89)	0 (4.98)	0.987 ^α^	0.959 ^α^
Perifoveal temporal quadrant DVD (%)	48.25 (5.43)	48.21 (5.91)	-0.06 (5.31)	0.923	0.959
Perifoveal superior quadrant SVD (%)	46.68 (4.38)	46.87 (4.69)	0.19 (4.79)	0.816 ^α^	0.959 ^α^
Perifoveal superior quadrant DVD (%)	45.03 (6.13)	45.25 (6.88)	0.07 (7.01)	0.934	0.959
Perifoveal nasal quadrant SVD (%)	51.22 (3.91)	51.13 (4.15)	-0.07 (5.03)	0.907	0.959
Perifoveal nasal quadrant DVD (%)	43.73 (6.71)	44.33 (6.24)	0.53 (8.17)	0.619	0.959
Perifoveal inferior quadrant SVD (%)	47.44 (4.8)	47.52 (4.72)	0.17 (5.26)	0.577 ^α^	0.959 ^α^
Perifoveal inferior quadrant DVD (%)	43.39 (6.15)	43.91 (6.28)	0.44 (7.11)	0.634	0.959
Whole image SVD (%)	46.31 (3.9)	46.11 (4.38)	-0.18 (4.17)	0.784 ^α^	0.959 ^α^
Whole image DVD (%)	44.58 (4.97)	44.47 (5.18)	-0.17 (5.15)	0.791	0.959
Whole image superior hemiretina SVD (%)	46.10 (3.77)	46.18 (4.21)	0.1 (4.2)	0.831 ^α^	0.959 ^α^
Whole image superior hemiretina DVD (%)	45.09 (5.14)	44.99 (5.81)	-0.18 (6)	0.817	0.959
Whole image inferior hemiretina SVD (%)	46.53 (4.21)	46.23 (4.4)	-0.27 (4.2)	0.656 ^α^	0.959 ^α^
Whole image inferior hemiretina DVD (%)	43.97 (5.13)	43.64 (5.1)	-0.37 (5.59)	0.607	0.959

Paired comparisons were done by paired t-test or Wilcoxon signed-rank test (denoted by α superscript), depending on the variables’ normality of distribution. All adjusted p-values of tests comparing perfusion indices were corrected for false discovery rate (FDR), using the Benjamini-Hochberg procedure. Identical adjusted p-values reflect the rank-based nature of the FDR correction

Abbreviations: DVD, deep vessel density; FAZ, foveal avascular zone; SVD, superficial vessel density

However, linear mixed effects models with different levels of adjustment supported no association between changes in serum 25(OH)D3 concentration and alterations in any of the macular perfusion parameters (Table 3). Likewise, changes in BCVA values (in LogMAR) were not associated with changes in serum 25(OH)D3 levels (coefficient = -0.001, p = 0.185). The difference in BCVA (LogMAR) was affected by baseline BCVA (coefficient=-0.231, p = 0.001) and, to a much smaller extent, by age (coefficient = 0.003, p = 0.012) and DM duration (coefficient = -0.003, p = 0.047).

**Table 3:** Association of changes in serum 25(OH)D_3_ levels with changes in angiographic outcome measures based on multiple linear mixed effects models for each outcome measure

Outcome measure	Model 1		Model 2		Model 3	
	Coefficient	95% CI	Coefficient	95% CI	Coefficient	95% CI
FAZ area difference	-0.00	-0.00–0.00	-0.00	-0.00–0.00	-0.00	-0.00–0.00
FAZ perimeter difference	-0.00	-0.02–0.01	-0.00	-0.02–0.01	-0.00	-0.02–0.01
Foveal SVD difference	0.04	-0.15–0.22	0.04	-0.16–0.24	-0.00	-0.26–0.26
Foveal DVD difference	-0.06	-0.34–0.21	-0.09	-0.38–0.20	-0.17	-0.52–0.19
Parafoveal SVD difference	-0.01	-0.22–0.19	-0.03	-0.25–0.19	-0.06	-0.30–0.18
Parafoveal DVD difference	-0.04	-0.23–0.16	-0.09	-0.29–0.11	-0.07	-0.29–0.15
Parafoveal superior hemiretina SVD difference	-0.10	-0.31–0.12	-0.11	-0.34–0.13	-0.13	-0.39–0.12
Parafoveal superior hemiretina DVD difference	-0.07	-0.25–0.12	-0.10	-0.30–0.09	-0.10	-0.34–0.15
Parafoveal inferior hemiretina SVD difference	0.05	-0.17–0.26	0.02	-0.21–0.26	-0.01	-0.28–0.27
Parafoveal inferior hemiretina DVD difference	0.01	-0.18–0.21	-0.06	-0.26–0.14	-0.05	-0.27–0.16
Parafoveal temporal quadrant SVD difference	-0.03	-0.25–0.19	-0.10	-0.32–0.12	-0.08	-0.34–0.17
Parafoveal temporal quadrant DVD difference	-0.02	-0.21–0.16	-0.10	-0.30–0.09	-0.13	-0.35–0.09
Parafoveal superior quadrant SVD difference	0.01	-0.23–0.26	0.00	-0.26–0.27	-0.00	-0.32–0.31
Parafoveal superior quadrant DVD difference	0.06	-0.15–0.27	0.03	-0.20–0.25	0.04	-0.24–0.31
Parafoveal nasal quadrant SVD difference	0.15	-0.08–0.38	0.14	-0.11–0.39	0.12	-0.19–0.44
Parafoveal nasal quadrant DVD difference	0.01	-0.20–0.22	-0.04	-0.26–0.17	-0.01	-0.27–0.25
Parafoveal inferior quadrant SVD difference	0.02	-0.21–0.25	-0.00	-0.25–0.25	-0.04	-0.34–0.26
Parafoveal inferior quadrant DVD difference	-0.05	-0.30–0.21	-0.10	-0.36–0.16	-0.08	-0.38–0.23
Perifoveal SVD difference	-0.01	-0.19–0.17	-0.09	-0.28–0.09	-0.11	-0.32–0.11
Perifoveal DVD difference	-0.00	-0.26–0.25	-0.05	-0.32–0.22	-0.02	-0.34–0.30
Perifoveal superior hemiretina SVD difference	-0.03	-0.21–0.14	-0.10	-0.28–0.08	-0.11	-0.32–0.11
Perifoveal superior hemiretina DVD difference	-0.02	-0.31–0.27	-0.08	-0.38–0.21	-0.05	-0.41–0.30
Perifoveal inferior hemiretina SVD difference	-0.00	-0.19–0.19	-0.09	-0.28–0.11	-0.09	-0.32–0.13
Perifoveal inferior hemiretina DVD difference	0.01	-0.25–0.27	-0.03	-0.30–0.25	0.01	-0.32–0.33
Perifoveal temporal quadrant SVD difference	-0.10	-0.30–0.10	-0.12	-0.33–0.10	-0.10	-0.37–0.16
Perifoveal temporal quadrant DVD difference	-0.04	-0.27–0.20	-0.06	-0.32–0.20	-0.09	-0.40–0.22
Perifoveal superior quadrant SVD difference	0.02	-0.18–0.22	-0.04	-0.25–0.16	-0.06	-0.30–0.18
Perifoveal superior quadrant DVD difference	0.05	-0.25–0.35	0.00	-0.32–0.32	0.03	-0.34–0.40
Perifoveal nasal quadrant SVD difference	-0.05	-0.24–0.15	-0.14	-0.33–0.04	-0.16	-0.36–0.04
Perifoveal nasal quadrant DVD difference	-0.05	-0.33–0.23	-0.09	-0.38–0.21	-0.04	-0.39–0.30
Perifoveal inferior quadrant SVD difference	0.03	-0.17–0.24	-0.05	-0.26–0.15	-0.05	-0.29–0.18
Perifoveal inferior quadrant DVD difference	0.03	-0.25–0.32	0.00	-0.30–0.31	0.07	-0.28–0.42
Whole image SVD difference	0.01	-0.17–0.19	-0.06	-0.25–0.14	-0.10	-0.33–0.12
Whole image DVD difference	-0.01	-0.23–0.21	-0.05	-0.28–0.18	-0.02	-0.28–0.25
Whole image superior hemiretina SVD difference	-0.02	-0.19–0.16	-0.08	-0.27–0.11	-0.10	-0.32–0.12
Whole image superior hemiretina DVD difference	-0.02	-0.28–0.23	-0.08	-0.34–0.18	-0.08	-0.41–0.24
Whole image inferior hemiretina SVD difference	0.03	-0.15–0.20	-0.04	-0.22–0.14	-0.08	-0.29–0.13
Whole image inferior hemiretina DVD difference	-0.01	-0.24–0.22	-0.06	-0.30–0.18	-0.07	-0.36–0.23

Abbreviations: DVD, deep vessel density; FAZ, foveal avascular zone; LME, linear mixed effects model; SVD, superficial vessel density

All models were adjusted for the baseline value of outcome measures, intra-individual correlation between eyes. Model 2 was additionally adjusted forage and gender. In addition to those factors, model 3 took into account diabetes mellitus duration, hemoglobin A1c level, diabetic retinopathy severity, and systolicblood pressure at baseline. None of the associations were statistically significant (all p-values were larger than 0.05)

## Discussion

We evaluated the impact of VDD correction on OCTA macular perfusion metrics in diabetic eyes with NDR, mild NPDR, and moderate NPDR. Our main findings include: (i) FAZ metrics did not change significantly, (ii) parafoveal SVD at the superior quadrant was slightly reduced, but the rest of the perfusion density indices remained unchanged, (iii) changes in BCVA showed a slight trend toward improvement, but it was not associated with changes in vitamin D levels, and (iv) changes in none of the studied OCTA metrics were associated with increases in serum 25(OH)D3 level.

Several observational studies have examined the association between VDD and DR. While observational studies have yielded inconsistent findings regarding the association between vitamin D status and diabetic retinopathy, numerous reports suggest an inverse relationship [27], whereas some demonstrate either no association or no significant difference between individuals with DR and healthy controls [28–31]. A meta-analysis by Trott et al. [32] found a significant association between VDD and sight-threatening DR (STDR), but not with non-sight-threatening DR (NSTDR). This suggests that the potential protective effects of vitamin D may be more evident in severe forms of DR, which aligns with literature indicating an inverse relationship between vitamin D levels and DR severity [33, 34]. However, Zhang et al. [33] found significant associations between vitamin D deficiency and DR, in both NPDR and PDR subgroups. Another systematic review by Chan et al. [35] summarized these discrepancies, highlighting that while vitamin D status may be associated with the presence of DR, it does not necessarily correlate with its severity. Differences in study design and population characteristics, such as retinopathy severity or the degree of vitamin D deficiency, may partly account for the inconsistencies across studies, alongside other confounding factors inherent to observational research.

From a pathogenetic standpoint, several pathways could explain the potential role of VDD in DR. Poor glycemic control is a recognized risk factor for DR, and optimal vitamin D levels are essential for insulin secretion, sensitivity, and function, which are crucial for glycemic control [25, 36, 37]. The anti-inflammatory and anti-angiogenesis properties of vitamin D [23, 38, 39], along with VDR activation’s ability to inhibit pro-inflammatory pathways in retinopathy [40], suggest its potential direct benefits of vitamin D, especially in later stages of DR, which involves upregulated pro-inflammatory cytokines [41]. It is possible that vitamin D insufficiency in diabetic patients may adversely affect glycemic control and exacerbate DR, as suggested by a population-based cohort, by Millen et al. [42], as they found that adjusting for HbA1c attenuated the odds ratio for DR, indicating a portion of the observed risk reduction in some patients with relatively high serum 25(OH)D may be caused by better glycemic control, not vitamin D levels itself. Similarly, two large prospective studies found no significant association between vitamin D levels and diabetic retinopathy after adjusting for factors like HbA1c, physical activity, and other confounders [43, 44].

A Mendelian randomization study by Huang et al. [14] did not find a significant causal relationship between serum 25(OH)D levels and the risk of DR. Their findings suggest that while some observational studies have suggested a link between vitamin D deficiency and DR, genetic data do not support a direct causal effect. This is consistent with another Mendelian Randomization analysis by Fan et al. [15] which also showed no causal relationship between vitamin D levels and most ocular diseases, including DR. These results highlight the complexity of interpreting observational data, demonstrating that findings from such studies may be significantly confounded by other variables such as glycemic control rather than accurately reflecting a true causal effect of vitamin D. In our cohort, most patients had HbA1c levels below 7, indicating good glycemic control [45]. These patients had no or minimal evidence of DR, where the role of angiogenesis is less prominent. Therefore, the minimal impact of VDD correction on retinal perfusion metrics in our study may reflect the minimal role of vitamin D in these early stages of DR.

Another factor that may contribute to discrepancies between studies is the definition of vitamin D deficiency and the target level achieved after correction. A pooled analysis by Yuan and colleagues [46] highlighted a non-linear relationship between declining vitamin D levels and DR, underscoring the significance of the chosen cutpoints. In our study, VDD was defined as serum vitamin D levels < 20 ng/ml, and after correction, serum levels reached > 30 in all patients, which may differ from other studies that used other thresholds [32, 33, 46]. Yuan et al.‘s study has also indicated that the risk of DR (overall) and PDR in individuals with VDD was increased compared to non-VDD individuals. A meta-analysis of 15 studies found an association between quantitative serum 25(OH)D levels and DR, but dichotomous categories of serum vitamin D 25(OH)D levels, i.e., below and above 30 ng/ml, were not significantly linked to DR. The association between vitamin D and the severity of DR was not analyzed in their study [47].

In our previous interventional study, we examined the effect of VDD correction in patients with diabetic macular edema (DME) undergoing IVB treatment, showing that it led to greater improvements in visual acuity and central macular thickness [17]. We found in that study that VDD correction could enhance the efficacy of IVB treatment in DME patients, and the present study is the first to investigate whether VDD correction exerts an angiographically-evident effect in eyes with non-severe diabetic retinopathy without DME. We observed a small but statistically significant reduction in superior parafoveal SVD. However, the absence of corresponding changes in other quadrants makes a true physiological effect unlikely and raises the possibility of measurement variability or signal underdetection. This spatially isolated pattern is also inconsistent with early diabetic retinopathy microvascular changes, which typically appear as more diffuse or regionally consistent alterations within the parafoveal capillary network. Taken together, this distribution further supports that the observed reduction most likely reflects measurement variability rather than a true disease-related structural change, as disease- or treatment-related effects would be expected to occur more uniformly across regions or rings [24]. Alternatively, it is also possible that such an anti-inflammatory effect of vitamin D exists, but inflammation was not severe and extensive enough in our patients for it to surface. That said, our linear mixed effects models did not support an association between increases in serum 25(OH)D_3_ levels and changes in any of the perfusion metrics, even the two that showed a slight reduction (Table 3). Changes in BCVA showed a slight trend toward improvement, but this was also not associated with changes in vitamin D levels. The slight trend toward VA improvement should be interpreted cautiously, as small VA changes may reflect test–retest or training effects rather than true functional improvement. This is further supported by the absence of corresponding changes in OCTA perfusion metrics and the lack of association with serum 25(OH)D₃ levels.

This study has several limitations. First, it lacked a control group of patients with DR and VDD who do not receive vitamin D supplements, making it difficult to directly compare outcomes. Second, the relatively short follow-up period of three months after VDD correction may not have been long enough to detect significant changes in perfusion metrics. Third, the small 6 × 6 field of view may have missed changes in more peripheral regions of the retina, where alterations might be more prominent in moderate and severe NPDR. Fourth, imaging was not strictly timed to control for diurnal variations, which may have introduced variability in OCTA measurements. [48]. Finally, although VA was selected as a standardized and clinically relevant functional outcome, it may be relatively insensitive to early neurovascular dysfunction in diabetes. More sensitive functional measures, such as contrast sensitivity, microperimetry, dark adaptation testing, or electrophysiologic assessments (e.g., mfERG), could better capture early functional alterations and should be incorporated in future studies.

In conclusion, this study suggests that correcting VDD has minimal impact on macular OCTA perfusion, if any, in people with diabetes and mild DR over a short period. Given the exploratory design and the short follow-up, these findings should be interpreted cautiously. Larger, longitudinal randomized controlled trials are needed to assess the role of vitamin D correction in DR progression. Future studies should also account for confounders like physical activity, which may influence DR risk [13], and consider incorporating more sensitive and precise functional measures to detect potential subtle changes in retinal function that may not be captured by standard visual acuity alone. While vitamin D may support ocular health, its direct effect on DR remains unclear, particularly in the early stages.

## Data Availability

The datasets generated and/or analyzed during the current study are available from the corresponding author on reasonable request.
